# Impact of cancer on outcomes following breakthrough ischaemic stroke on oral anticoagulants for atrial fibrillation: insights from the ASPERA-R study

**DOI:** 10.1093/esj/aakag015

**Published:** 2026-02-27

**Authors:** Matteo Foschi, Federico De Santis, Francesca Gabriele, Lucio D’Anna, Andrea Zini, Matteo Paolucci, Stefano Forlivesi, Ludovica Migliaccio, Maria Maddalena Viola, Angelo Cascio Rizzo, Maria Sessa, Ghil Schwarz, Rachele Tortorella, Soma Banerjee, Gaurav Desai, Muhammad Jaffar, Gabriele Prandin, Leonardo Pantoni, Francesco Mele, Giuseppe Scopelliti, Ilaria Cova, Mariarosaria Valente, Domenico Maisano, Luca Antonelli, Maria Rosaria Bagnato, Giovanni Di Mauro, Francesca Bernocchi, Martina Gaia Di Donna, Barbara Casolla, Myriam Perla Mazloum, Kristina Kacani, Noufel-Anis Djeghlal, Laura González-Martín, Ricardo Rigual, Blanca Fuentes, Carlos Hervás, Paolo Candelaresi, Vincenzo Andreone, Antonio De Mase, Emanuele Spina, Diana Aguiar de Sousa, Mariana Almudi Souza, Alberto Fior, Miguel Serôdio, Pietro Caliandro, Aurelia Zauli, Giuseppe Reale, Ahmed Abdelalim, Sandra Ahmed, Nourhan Mohamed Soliman, Liqun Zhang, Tara Latimer, Muhammad Elboghday, Ahmed Aly Elbassiouny, Tamer Roushdy, Hossam Shokri, Federica Ferrari, Nicola Davide Loizzo, Federico Mazzacane, Maria Guarino, Valentina Barone, Paola Forti, Giuseppe Rinaldi, Marco Vito Rossi, Vincenzo Laterza, Giovanni Frisullo, Pier Andrea Rizzo, Aldobrando Broccolini, Marina Mannino, Valeria Terruso, Marcella Caggiula, Annalisa Rizzo, Ana Catarina Fonseca, Bernardo Antunes, Ana M Barbosa, Hrvoje Budincevic, Petra Crnac, Giovanna Viticchi, Mauro Silvestrini, Lorenzo Barba, Markus Otto, Piergiorgio Lochner, Benjamin Landau, Sandeep Buddha, Roumeisa Khalil, Maria Grazia Piscaglia, Elena Minguzzi, Marialuisa Zedde, Ahmed Nasreldein, Luisa Vinciguerra, Luis Costa, Ahmed Elsaid Elsayed, Mona AlBanna, Laura Tudisco, Maria Giulia Mosconi, Giovanni Merlino, Alexandros Polymeris, Raffaele Ornello, Simona Sacco

**Affiliations:** Department of Biotechnological and Applied Clinical Sciences, University of L’Aquila, L’Aquila, Italy; Department of Neurosciences, Stroke Unit—Neurology Unit, S.Maria delle Croci Hospital, AUSL Romagna, Ravenna, Italy; Department of Biotechnological and Applied Clinical Sciences, University of L’Aquila, L’Aquila, Italy; Department of Biotechnological and Applied Clinical Sciences, University of L’Aquila, L’Aquila, Italy; Department of Stroke and Neuroscience, Charing Cross Hospital, Imperial College NHS Healthcare Trust, London, United Kingdom; Department of Brain Sciences, Imperial College London, London, United Kingdom; Department of Neurology and Stroke Center, Maggiore Hospital, IRCCS Istituto delle Scienze Neurologiche di Bologna, Bologna, Italy; Department of Neurology and Stroke Center, Maggiore Hospital, IRCCS Istituto delle Scienze Neurologiche di Bologna, Bologna, Italy; Department of Neurology and Stroke Center, Maggiore Hospital, IRCCS Istituto delle Scienze Neurologiche di Bologna, Bologna, Italy; Department of Neurology and Stroke Center, Maggiore Hospital, IRCCS Istituto delle Scienze Neurologiche di Bologna, Bologna, Italy; Department of Neurology and Stroke Center, Maggiore Hospital, IRCCS Istituto delle Scienze Neurologiche di Bologna, Bologna, Italy; Department of Neurology and Stroke Unit, ASST Grande Ospedale Metropolitano Niguarda, Milan, Italy; Department of Neurology and Stroke Unit, ASST Grande Ospedale Metropolitano Niguarda, Milan, Italy; Department of Neurology and Stroke Unit, ASST Grande Ospedale Metropolitano Niguarda, Milan, Italy; Department of Neurology and Stroke Unit, ASST Grande Ospedale Metropolitano Niguarda, Milan, Italy; Department of Stroke and Neuroscience, Charing Cross Hospital, Imperial College NHS Healthcare Trust, London, United Kingdom; Department of Stroke and Neuroscience, Charing Cross Hospital, Imperial College NHS Healthcare Trust, London, United Kingdom; Department of Stroke and Neuroscience, Charing Cross Hospital, Imperial College NHS Healthcare Trust, London, United Kingdom; Clinical Unit of Neurology, Department of Medicine, Surgery and Health Sciences, University Hospital and Health Services of Trieste, ASUGI, University of Trieste, Trieste, Italy; Neuroscience Research Center, Department of Biomedical and Clinical Sciences, University of Milan, Milan, Italy; Department of Neurorehabilitation Sciences, Casa di Cura Igea, Milan, Italy; Neurology Unit, University Hospital Luigi Sacco, Milan, Italy; Neurology Unit, University Hospital Luigi Sacco, Milan, Italy; Neurology Unit, University Hospital Luigi Sacco, Milan, Italy; Clinical Neurology, DMED, University of Udine, Udine, Italy; Clinical Neurology, DMED, University of Udine, Udine, Italy; Clinical Neurology, DMED, University of Udine, Udine, Italy; UOC Stroke Unit e Neurologia, Ospedale Fabrizio Spaziani, Frosinone, Italy; UOC Stroke Unit e Neurologia, Ospedale Fabrizio Spaziani, Frosinone, Italy; UOC Stroke Unit e Neurologia, Ospedale Fabrizio Spaziani, Frosinone, Italy; UOC Stroke Unit e Neurologia, Ospedale Fabrizio Spaziani, Frosinone, Italy; Stroke Unit, CHU Pasteur 2, Université Cote d'Azur, UMR2CA, Nice, France; Stroke Unit, CHU Pasteur 2, Université Cote d'Azur, UMR2CA, Nice, France; Stroke Unit, CHU Pasteur 2, Université Cote d'Azur, UMR2CA, Nice, France; Stroke Unit, CHU Pasteur 2, Université Cote d'Azur, UMR2CA, Nice, France; Stroke Center and Department of Neurology, Hospital La Paz Institute for Health Research-ldlPAZ (La Paz University Hospital-Universidad Autónoma de Madrid), Madrid, Spain; Stroke Center and Department of Neurology, Hospital La Paz Institute for Health Research-ldlPAZ (La Paz University Hospital-Universidad Autónoma de Madrid), Madrid, Spain; Stroke Center and Department of Neurology, Hospital La Paz Institute for Health Research-ldlPAZ (La Paz University Hospital-Universidad Autónoma de Madrid), Madrid, Spain; Stroke Center and Department of Neurology, Hospital La Paz Institute for Health Research-ldlPAZ (La Paz University Hospital-Universidad Autónoma de Madrid), Madrid, Spain; UOC Neurologia e Stroke Unit, AORN Cardarelli, Napoli, Italy; UOC Neurologia e Stroke Unit, AORN Cardarelli, Napoli, Italy; UOC Neurologia e Stroke Unit, AORN Cardarelli, Napoli, Italy; UOC Neurologia e Stroke Unit, AORN Cardarelli, Napoli, Italy; Stroke Center, Department of Neurosciences, Centro Hospitalar Universitário Lisboa Central, ULS São José, and Faculdade de Medicina, Universidade de Lisboa, Lisbon, Portugal; Stroke Center, Department of Neurosciences, Centro Hospitalar Universitário Lisboa Central, ULS São José, Lisbon, Portugal; Stroke Center, Department of Neurosciences, Centro Hospitalar Universitário Lisboa Central, ULS São José, Lisbon, Portugal; Stroke Center, Department of Neurosciences, Centro Hospitalar Universitário Lisboa Central, ULS São José, Lisbon, Portugal; Department of Neuroscience, Catholic University of the Sacred Hearth, Rome, Italy; UOC Neurology, Department of Neuroscience, Sensory Organs, and Thorax, Fondazione Policlinico Universitario A. Gemelli IRCCS, Rome, Italy; Department of Neuroscience, Catholic University of the Sacred Hearth, Rome, Italy; UOC Neurology, Department of Neuroscience, Sensory Organs, and Thorax, Fondazione Policlinico Universitario A. Gemelli IRCCS, Rome, Italy; UOC Neuroriabilitazione ad Alta Intensità, Fondazione Policlinico Universitario A. Gemelli IRCCS, Rome, Italy; Cairo University Stroke Center, Department of Neurology, Faculty of Medicine, Cairo University, Giza, Egypt; Cairo University Stroke Center, Department of Neurology, Faculty of Medicine, Cairo University, Giza, Egypt; Cairo University Stroke Center, Department of Neurology, Faculty of Medicine, Cairo University, Giza, Egypt; Department of Neurology, St George’s University Hospital, London, United Kingdom; Department of Neurology, St George’s University Hospital, London, United Kingdom; Department of Neurology, St George’s University Hospital, London, United Kingdom; Neurology Department, Faculty of Medicine, Ain Shams University, Cairo, Egypt; Neurology Department, Faculty of Medicine, Ain Shams University, Cairo, Egypt; Neurology Department, Faculty of Medicine, Ain Shams University, Cairo, Egypt; Department of Brain and Behavioral Sciences, University of Pavia, Pavia, Italy; Department of Emergency Neurology and Stroke Unit, IRCCS Mondino Foundation, Pavia, Italy; Department of Emergency Neurology and Stroke Unit, IRCCS Mondino Foundation, Pavia, Italy; Department of Brain and Behavioral Sciences, University of Pavia, Pavia, Italy; Department of Emergency Neurology and Stroke Unit, IRCCS Mondino Foundation, Pavia, Italy; IRCCS Istituto delle Scienze Neurologiche di Bologna, Bologna, Italy; IRCCS Istituto delle Scienze Neurologiche di Bologna, Bologna, Italy; Department of Medical and Surgical Sciences, University of Bologna, IRCCS Azienda Ospedaliero-Universitaria di Bologna, Bologna, Italy; S.C. Neurologia, Ospedale “Di Venere”, Bari, Italy; Department of Neurosciences, Stroke Unit—Neurology Unit, S.Maria delle Croci Hospital, AUSL Romagna, Ravenna, Italy; Neuroscience Research Center, Department of Biomedical and Clinical Sciences, University of Milan, Milan, Italy; S.C. Neurologia, Ospedale “Di Venere”, Bari, Italy; Emergency Neurology Unit, Department of Neuroscience, Sensory Organs, and Thorax, Policlinico Universitario Agostino Gemelli IRCCS, Rome, Italy; Catholic University of the Sacred Heart, Fondazione Policlinico Agostino Gemelli IRCCS, Rome, Italy; Stroke Unit, Department of Neuroscience, Sensory Organs, and Thorax, Policlinico Universitario Agostino Gemelli IRCCS, Rome, Italy; UOC Neurologia e Stroke Unit, AOOR Villa Sofia-Cervello, Palermo, Italy; UOC Neurologia e Stroke Unit, AOOR Villa Sofia-Cervello, Palermo, Italy; UOC Neurologia e Stroke Unit, PO Vito Fazzi, Lecce, Italy; UOC Neurologia e Stroke Unit, PO Vito Fazzi, Lecce, Italy; Neurology Department, Hospital de Santa Maria, Faculdade de Medicina, University of Lisbon, Lisbon, Portugal; Department of Neurology, Hospital de Santa Maria, Lisbon, Portugal; Department of Neurology, Hospital de Santa Maria, Lisbon, Portugal; Department of Neurology, Sveti Duh University Hospital, Zagreb, Croatia; Department of Neurology and Neurosurgery, Faculty of Medicine, J.J. Strossmayer University of Osijek, Osijek, Croatia; Department of Psychiatry and Neurology, Faculty of Dental Medicine and Health, J.J. Strossmayer University of Osijek, Osijek, Croatia; Department of Neurology, Sveti Duh University Hospital, Zagreb, Croatia; Neurological Clinic, Marche Polytechnic University, Ancona, Italy; Neurological Clinic, Marche Polytechnic University, Ancona, Italy; Department of Neurology, Martin-Luther-University Halle-Wittenberg, Halle (Saale), Germany; Department of Neurology, Martin-Luther-University Halle-Wittenberg, Halle (Saale), Germany; Department of Neurology, Saarland University Medical Center, Homburg 66421, Germany; Department of Neurology, Saarland University Medical Center, Homburg 66421, Germany; Department of Stroke Medicine, Southmead Hospital, North Bristol NHS Trust, Bristol, United Kingdom; Department of Stroke Medicine, Southmead Hospital, North Bristol NHS Trust, Bristol, United Kingdom; Department of Neurosciences, Stroke Unit—Neurology Unit, S.Maria delle Croci Hospital, AUSL Romagna, Ravenna, Italy; Department of Neurosciences, Stroke Unit—Neurology Unit, S.Maria delle Croci Hospital, AUSL Romagna, Ravenna, Italy; Neurology Unit, Stroke Unit, Azienda Unità Sanitaria Locale-IRCCS di Reggio Emilia, Reggio Emilia, Italy; Department of Neurology, Assiut University, Assiut, Egypt; Department of Neurology and Stroke Unit, ASST Crema Hospital, Crema, Italy; Department of Neurology, Local Health Unit of Alto Minho, Viana do Castelo, Portugal; Neurology and Neurointervention Department, Kobry Elkoba Medical Complex, Cairo, Egypt; Department of Neurology, National Neuroscience Institute, King Fahad Medical City, Riyadh, Saudi Arabia; Stroke Unit, Careggi University Hospital, Florence, Italy; Department of Internal and Cardiovascular Medicine, Santa Maria della Misericordia Hospital, Perugia, Italy; SOSD Stroke Unit, Udine University Hospital, Udine, Italy; Stroke Division, Department of Neurology, Beth Israel Deaconess Medical Center, Harvard Medical School, Boston, MA, United States; Department of Neurology and Stroke Center, University Hospital Basel and University of Basel, Basel, Switzerland; Department of Biotechnological and Applied Clinical Sciences, University of L’Aquila, L’Aquila, Italy; Department of Biotechnological and Applied Clinical Sciences, University of L’Aquila, L’Aquila, Italy

**Keywords:** atrial fibrillation, cancer, ischaemic stroke, malignancy, oral anticoagulation, outcomes, prognosis

## Abstract

**Background:**

Breakthrough ischaemic stroke during oral anticoagulation (OAC) for atrial fibrillation (AF) represents a major therapeutic challenge, especially in patients with cancer, who face competing risks of thrombosis and bleeding. This study investigated the impact of cancer on 90-day outcomes after ischaemic stroke on OAC.

**Patients and methods:**

We analysed patients with AF who experienced ischaemic stroke while on continuous OAC enrolled in the international retrospective ASPERA-R study, comprising 35 stroke centres across 9 countries. Inverse probability weighting was applied to adjust for baseline imbalances, and weighted Cox, ordinal logistic and generalised linear models were used to estimate adjusted 90-day risks for the primary (ischaemic stroke or TIA), secondary (mRS shift, vascular/all-cause death) and safety (moderate-to-severe bleeding, ICH, 24-h haemorrhagic transformation) outcomes.

**Results:**

Among 1649 included patients (mean age 78.0 ± 10.7 years; 45.8% male), 247 (15.0%) had cancer, of whom 87 (35.2%) had active cancer and 160 (64.8%) were in remission. After weighting, patients with cancer had a significantly higher 90-day risk of new ischaemic stroke or TIA (8.2% vs 2.8%; adjusted hazard ratio [aHR] 2.56; 95% CI, 1.59–4.13; *P* < .001) and worse 90-day mRS score distribution (adjusted odds ratio 1.29; 95% CI, 1.08–1.54; *P* = .005) than those without cancer. Active cancer conferred a > 4-fold higher risk of new ischaemic stroke or TIA (HR 4.48, 95% CI, 2.46–8.13; *P* < .001) and nearly 3-fold higher risk of moderate-to-severe bleeding (HR 2.77; 95% CI, 1.30–5.88; *P* = .008). Patients with cancer in remission showed increased ischaemic risk (HR 2.60; 95% CI, 1.59–5.25; *P* = .001) but not bleeding risk. Haematological malignancies carried a higher risk for both new ischaemic stroke or TIA (HR 3.06; 95% CI, 1.69–5.54; *P* = .001) and moderate-to-severe bleeding (HR 3.47, 95% CI, 1.57–7.70; *P* = .006) compared to solid malignancies.

**Conclusion:**

Cancer, particularly active and haematological malignancies, substantially worsens 90-day prognosis after breakthrough stroke on OAC, underscoring the need for refined risk stratification and tailored secondary prevention.

**Trial registration:**

URL: www.clinicaltrials.gov; Unique identifier: NCT06823466.

## Graphical Abstract

**Graphical Abstract:**
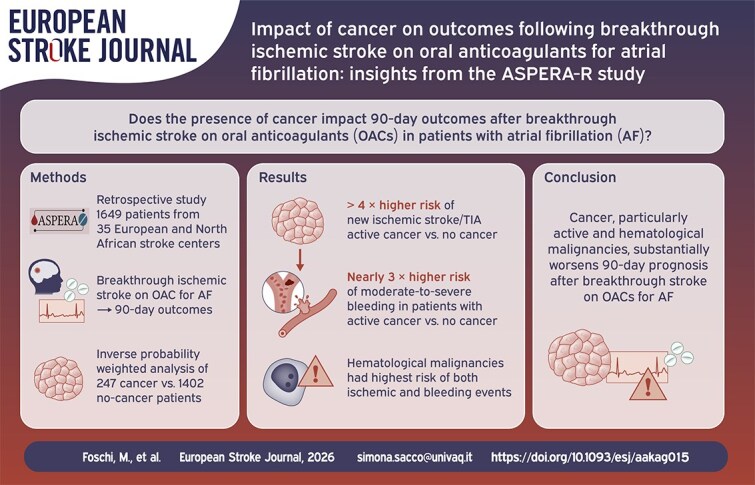

## Introduction

Over the past decades, major advances in atrial fibrillation (AF) management have substantially improved stroke prevention. Oral anticoagulation (OAC), particularly with the advent of direct oral anticoagulants (DOACs), represents the cornerstone of secondary prevention, significantly reducing ischaemic stroke risk.^1,2^ However, a subset of patients still experiences breakthrough ischaemic strokes despite adequate OAC, remaining at high risk of recurrence. A recent patient-level meta-analysis estimated this residual annual risk at 1.5%–2.5%.^3^ These individuals constitute a distinct and particularly vulnerable phenotype, facing an increased risk of both recurrent ischaemic events and major bleeding, underscoring the need for tailored preventive strategies.^4,5^

In patients with ischaemic stroke, concomitant cancer introduces a therapeutic paradox in which tumour-driven hypercoagulability, endothelial injury and treatment-related haemostatic alterations^6–8^ simultaneously promote thrombosis and predispose to bleeding. In AF patients receiving OAC, this hypercoagulable state may counteract anticoagulant efficacy, increasing the likelihood of breakthrough ischaemia, while cancer and its treatments further heighten bleeding risk.^9,10^ Despite these challenges, breakthrough ischaemic strokes during OAC remain poorly investigated in patients with cancer. Clarifying their characteristics and clinical outcomes is crucial to guide evidence-based management and optimise secondary prevention in this complex population.

Hence, we aimed to assess the impact of cancer on outcomes following breakthrough ischaemic stroke in AF patients on continuous OAC therapy.

## Patients and methods

### Data availability

The dataset supporting this study will be made available upon reasonable request to the corresponding author.

### Study design

ASPERA is a large multicentre observational study coordinated by the University of L’Aquila, comprising a retrospective (ASPERA-R) and a prospective (ASPERA-P) arm. This analysis reports data from ASPERA-R, including 35 stroke centres across 9 European and North African countries (Appendix S1). The ASPERA-P arm is ongoing. The study followed STROBE reporting guidelines.^11^

### Study population

ASPERA-R consecutively enrolled adults (≥18 years) with AF who experienced a breakthrough ischaemic stroke while on continuous OAC between February 2020 and February 2025. All hospital admissions and emergency evaluations during this period were screened. Stroke diagnosis followed WHO criteria and was confirmed by NCCT or MRI. Continuous OAC use was confirmed based on prescription records and corroborated by patient or caregiver report of uninterrupted treatment during the 7 days prior to the index event, with documented last intake within 48 h of stroke onset.

### Data collection and definitions

Clinical data were retrieved from hospital records and standardised follow-up visits, supplemented by direct contact when needed. Only patients with complete 90-day follow-up and no missing mandatory baseline variables were included. The full list of both mandatory and optional baseline variables is provided in Appendix S2. Data were entered via a standardised REDCap case report form and centrally validated through weekly quality checks. The final dataset was locked on 1 September 2025.

Patients were classified as with or without cancer. Those with cancer were further categorised as active or in remission. Active cancer was defined using binary clinical information available in the registry (yes/no): (1) diagnosis within 6 months before or during the index hospitalisation; (2) receipt of cancer-directed therapy (radiotherapy, chemotherapy, hormonal therapy or surgery) within 6 months or (3) recurrence or metastasis within 6 months. Cancer in remission referred to a prior malignancy not fulfilling active cancer criteria. Importantly, the ASPERA-R registry did not collect detailed oncological data such as cancer stage (eg, tumour–node–metastasis [TNM] classification), specific treatment regimens, treatment timing/intensity or extent of metastatic disease. Cancer status was determined by the treating physician; no systematic screening for occult malignancies was performed.

### Study outcomes

The primary outcome was the 90-day risk of new ischaemic stroke or TIA. Secondary outcomes included the 90-day ordinal shift in mRS score; 90-day incidence all-cause and vascular death (any death attributable to cardiovascular or cerebrovascular causes, including fatal ischaemic or haemorrhagic stroke, sudden cardiac death, myocardial infarction or other vascular events). Safety outcomes included 90-day moderate-to-severe bleeding, ICH and 24-h haemorrhagic transformation (HT). Bleeding severity was categorised according to the Global Utilization of Streptokinase and Tissue Plasminogen Activator for Occluded Coronary Artery (GUSTO) trial classification.^12^ Specifically, moderate-to-severe bleeding was defined as any haemorrhagic event ranging from those requiring blood transfusion to life-threatening or fatal bleeding, including ICH or bleeding necessitating surgical intervention, fluid replacement or inotropic support. Haemorrhagic transformation was defined according to the Heidelberg Bleeding Classification system.^13^

### Statistical analysis

Categorical variables were expressed as counts (percentages) and continuous variables as means (SD) or medians (IQR). To minimise baseline imbalances between groups, inverse probability weighting (IPW) based on propensity scores was applied using logistic regression including prespecified variables (age, sex, ethnicity, enrolling centre, vascular risk factors, AF type, baseline NIHSS and mRS scores, concomitant large-artery atherosclerosis and reperfusion therapies). Stabilised weights were used to limit extreme values, and covariate balance was verified through standardised mean differences (SMDs) and visual inspection of score covariate balance graphs. Primary and secondary analyses were performed in the weighted cohort, with further adjustment for residual imbalances. The primary outcome was evaluated with weighted Cox regression models, providing adjusted hazard ratios (aHRs) and 95% CIs. Secondary outcomes were analysed with ordinal logistic regression for 90-day mRS and weighted Cox models for mortality. Safety endpoints (24-h haemorrhagic transformation, 90-day bleeding and ICH) were assessed using generalised linear or Cox models. Death was treated as a competing risk for recurrent ischaemic events, with cumulative incidences compared by Gray’s test and Fine–Gray regression. Proportional hazards assumptions were checked via Schoenfeld residuals, and Kaplan–Meier curves illustrated time-to-event outcomes. All eligible patients were included without formal sample size estimation. Analyses were conducted in R (v4.4.1), with 2-sided *P* < .05 considered significant.

## Results

**Figure 1:**
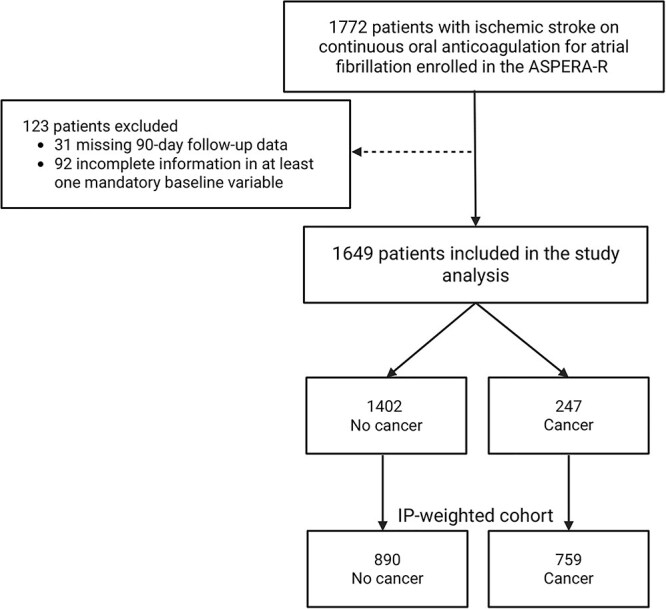
Study flow-chart of the ASPERA-R study. Abbreviations: IP = inverse probability.

A total of 1649 patients were included, of whom 247 (15.0%) had cancer (mean age 78.0 ± 10.7 years). The ASPERA-R flowchart with excluded cases is shown in Figure 1. Among cancer patients, 87/247 (35.2%) had active cancer and 160/247 (64.8%) cancer in remission. Cancer sites were gastrointestinal 48/247 (19.4%), lung 20/247 (8.1%), genitourinary 64/247 (25.9%), breast 47/247 (19.0%), haematological 30/247 (12.2%), skin 15/247 (6.1%) and other 23/247 (9.3%). Metastatic disease was present in 29/247 (11.7%), involving lymph nodes in 7/29 (24.1%), bone 5/29 (17.2%), liver 5/29 (17.2%), lung 5/29 (17.2%) and multiple sites 3/29 (10.3%). No patient had a primary or secondary tumour of the central nervous system. Unweighted characteristics are summarised in Table 1.

**Table 1:** Baseline characteristics.

	**Overall cohort (*n* = 1,649)**	**Cancer (*n* = 247)**	**No cancer (*n* = 1,402)**	**SMD unweighted**	**SMD weighted**
***Demographics***					
**Age (years), mean ± SD**	78.0 ± 10.7	79.4 ± 7.9	77.7 ± 10.9	0.263	**0.168**
**Sex—males, *n* (%)**	789 (47.8)	129 (52.2)	660 (47.1)	0.103	0.024
**Ethnicity, *n* (%)**				0.437	**0.395**
** Non-Hispanic White**	1,303 (79.0)	213 (86.2)	1,090 (77.7)		
** Hispanic White**	113 (6.9)	23 (9.3)	90 (6.4)		
** Black**	24 (1.5)	1 (0.4)	23 (1.6)		
** Asian**	9 (0.5)	1 (0.4)	8 (0.6)		
** Other**	200 (12.1)	9 (3.6)	191 (13.6)		
***Baseline oral anticoagulation characteristics***					
**Type of oral anticoagulation at the time of index stroke, *n* (%)**				0.012	0.036
** VKA**	374 (22.7)	55 (22.3)	319 (22.8)		
** DOAC**	1,275 (77.3)	192 (77.7)	1,083 (77.2)		
**Type of DOAC at the time of index stroke, *n* (%)**				0.053	0.074
** Apixaban**	469/1,275 (36.8)	71/192 (37.0)	398/1,083 (36.7)		
** Rivaroxaban**	428/1,275 (33.6)	57/192 (29.7)	371/1,083 (34.3)		
** Edoxaban**	251/1,275 (19.7)	43/192 (22.4)	208/1,083 (19.2)		
** Dabigatran**	127/1,275 (10.0)	21/192 (10.9)	106/1,083 (9.8)		
**Time from last DOAC intake to admission, *n* (%)**				0.208	0.098
** < 12 hours**	653/1,275 (51.2)	111/192 (57.8)	542/1,083 (50.0)		
** 12-24 hours**	471/1,275 (36.9)	68/192 (35.4)	403/1,083 (37.2)		
** 24-48 hours**	151/1,275 (11.9)	13/192 (6.8)	138/1,083 (12.8)		
**INR on admission, mean ± SD**	1.39 ± 0.54	1.40 ± 0.56	1.37 ± 0.45	0.051	0.032
**INR on admission—patients on VKAs, *n* (%)**				0.030	0.080
** < 2**	205/374 (54.8)	29/55 (52.7)	176/319 (55.2)		
** 2–3.5**	156/374 (41.7)	26/55 (47.3)	130/319 (40.8)		
** > 3.5**	13/374 (3.5)	0/55 (0.0)	13/319 (4.0)		
**DOAC levels on admission available, *n* (%)**	281/1,275 (22.0)	53/192 (27.6)	228/1,083 (21.1)	0.153	0.081
**DOAC levels on admission, *n* (%)**					
** Below range**	67/281 (23.9)	11/53 (20.8)	56/228 (24.6)	0.065	0.004
** Within range**	197/281 (70.1)	39/53 (73.6)	158/228 (69.3)		
** Above range**	17/281 (6.0)	3/53 (5.6)	14/228 (6.1)		
***Clinical characteristics***					
**Hospitalisation, *n* (%)**	1,596 (96.8)	240 (97.2)	1,356 (96.7)	0.026	0.003
**Hospital setting, *n* (%)**				0.018	0.043
** Stroke unit**	1,504/1,596 (94.2)	228/240 (95.0)	1276/1,356 (94.1)		
** Intensive care unit**	42/1,596 (2.6)	4/240 (1.7)	38/13,563 (2.8)		
** Other hospital unit**	50/1,596 (3.1)	8/240 (3.3)	42/1,356 (3.1)		
**NIHSS on admission, median (IQR)**	11 (5–18)	11 (5–18)	10 (5–17)	0.105	0.069
**Pre-stroke mRS score category, *n* (%)**				0.217	**0.210**
** No symptoms (score of 0), *n* (%)**	814 (49.4)	105 (42.5)	709 (50.6)		
** Symptoms without any disability (score of 1), *n* (%)**	369 (22.4)	58 (23.5)	311 (22.2)		
** Symptoms with mild disability (score of 2), *n* (%)**	223 (13.5)	41 (16.6)	182 (13.0)		
** Symptoms with mild-to-moderate disability (score of 3), *n* (%)**	179 (10.9)	32 (14.2)	144 (10.3)		
** Symptoms with moderate-to-severe disability (score of 4), *n* (%)**	59 (3.6)	8 (3.2)	51 (3.6)		
** Symptoms with severe disability (score of 5), *n* (%)**	5 (0.3)	0 (0.0)	5 (0.4)		
**Concomitant large-artery atherosclerosis, *n* (%)^a^**	240 (14.6)	23 (9.3)	217 (15.4)	0.186	0.097
**Intravenous thrombolysis, *n* (%)**	139 (8.4)	18 (7.3)	121 (8.6)	0.050	0.027
**Endovascular thrombectomy, *n* (%)**	732 (44.4)	110 (44.5)	622 (44.4)	0.003	0.019
***Risk factors***					
**Arterial hypertension, *n* (%)^b^**	1,338 (81.1)	200 (81.0)	1,138 (81.2)	0.003	0.026
**Dyslipidemia, *n* (%)^c^**	845 (51.2)	120 (48.6)	725 (51.7)	0.063	0.062
**Diabetes, *n* (%)^d^**	443 (26.9)	49 (19.8)	394 (28.1)	0.194	0.011
**Cigarette smoking, *n* (%)^e^**	154 (9.3)	20 (8.1)	134 (9.6)	0.051	0.058
**Prior ischaemic stroke or TIA, *n* (%)**	410 (24.9)	60 (24.3)	350 (25.0)	0.016	0.050
**Prior ICH, *n* (%)**	27 (1.6)	6 (2.4)	21 (1.5)	0.067	0.067
**Ischaemic heart disease, *n* (%)^f^**	366 (22.2)	57 (23.1)	309 (22.0)	0.025	0.088
**Chronic congestive heart failure, *n* (%)^g^**	277 (16.8)	41 (16.6)	236 (16.8)	0.006	0.052
**Chronic kidney disease, *n* (%)^h^**	268 (16.3)	48 (19.4)	220 (15.7)	0.098	0.018
**Chronic liver failure, *n* (%)^i^**	7 (0.4)	1 (0.4)	6 (0.4)	0.004	0.001
**Symptomatic peripheral artery disease, *n* (%)^j^**	80 (4.9)	16 (6.5)	64 (4.6)	0.084	0.024
**Mechanical heart valve, *n* (%)**	94 (5.7)	15 (6.1)	79 (5.6)	0.019	0.031
**Biological heart valve, *n* (%)**	81 (4.9)	19 (7.7)	62 (4.4)	0.137	0.006
**Atrial fibrillation type, *n* (%)^k^**				0.378	**0.267**
** Paroxysmal**	335 (20.3)	53 (21.5)	282 (20.1)		
** Persistent**	200 (12.1)	9 (3.6)	191 (13.6)		
** Long-standing persistent**	76 (4.6)	9 (3.6)	67 (4.8)		
** Permanent**	847 (51.4)	148 (59.9)	699 (49.9)		
** Unknown**	191 (11.6)	28 (11.3)	163 (11.6)		
**Antihypertensive drugs on admission, *n* (%)**	1,310 (79.4)	194 (78.5)	1,116 (79.6)	0.026	0.092
**Lipid-lowering drugs on admission, *n* (%)**	742 (45.0)	111 (44.9)	631 (45.0)	0.001	0.001
**Antidiabetic drugs on admission, *n* (%)**	397 (24.1)	44 (17.8)	353 (25.2)	0.180	0.015
**Antiplatelet therapy on admission, *n* (%)**	131 (7.9)	18 (7.3)	113 (8.1)	0.029	0.039
***Post-stroke secondary prevention***					
**Post-stroke anticoagulation strategy, *n* (%)**				0.164	**0.128**
** No anticoagulation restarted^l^**	179 (10.9)	25 (10.1)	179 (12.8)		
** Same DOAC restarted**	463 (28.1)	62 (25.1)	463 (33.0)		
** Switch to different DOAC within the FXa inhibitors class**	201 (12.2)	23 (9.3)	201 (14.3)		
** Switch to different DOAC (FXa inhibitors ↔ FIIa inhibitor)**	283 (17.2)	42 (17.0)	283 (27.2)		
** VKA → DOAC**	112 (6.8)	18 (7.9)	112 (8.0)		
** VKA → VKA**	179 (10.9)	27 (7.3)	179 (12.8)		
** DOAC → VKA**	59 (3.6)	16 (5.3)	59 (4.2)		
** DOAC → LMWH**	36 (2.2)	11 (4.5)	36 (2.6)		
** VKA → LMWH**	19 (1.2)	3 (1.2)	19 (1.6)		
** Left atrial appendage closure**	8 (0.5)	0 (0.0)	8 (0.6)		
** Unknown**	110 (6.7)	20 (8.1)	110 (7.8)		
**Days from index stroke to anticoagulation restart, mean ± SD**	8.1 ± 9.1	8.6 ± 9.9	8.0 ± 8.9	0.057	0.031
**Anticoagulation discontinued during follow-up, *n* (%)**	151/1,350 (11.2)	30/201 (11.5)	121/1,149	0.059	0.043
**Post-stroke antihypertensive drugs, *n* (%)**	1,346 (81.7)	208 (84.2)	1,138 (81.2)	0.080	0.096
**Post-stroke lipid-lowering drugs, *n* (%)**	1,064 (64.5)	161 (65.2)	903 (64.4)	0.007	0.018
**Post-stroke antidiabetic drugs, *n* (%)**	387 (23.5)	43 (17.4)	344 (24.5)	0.195	0.037
**Post-stroke antiplatelet therapy, *n* (%)**	250 (15.2)	38 (15.4)	212 (15.2)	0.001	0.018

^a^Concomitant large-artery atherosclerosis was classified according to the Trial of Org 10172 in the Acute Stroke Treatment (TOAST) classification system.

^b^History of blood pressure > 140/90 mmHg or the current use of antihypertensive medications.

^c^History of total blood cholesterol levels > 220 mg/dL and/or total triglycerides levels > 130 mg/dL and/or current used lipid-lowering drugs.

^d^History of fasting glucose > 126 mg/dL or the current use of hypoglycaemic medications.

^e^Consumption of ≥ 1 cigarette per day over the last year.

^f^Ischaemic heart disease was defined as history of myocardial infarction, angina or prior evidence of coronary disease on coronary angiography.

^g^History of stage C (structural heart disease and current or past history of heart-failure symptoms) or stage D (refractory symptoms that interfere with daily life or recurrent hospitalisation despite targeted guideline-directed medical therapy) chronic heart failure.

^h^History of estimated creatinine clearance of less than 60 for 3 months or more (including dialysis).

^i^History of cirrhosis or end-stage liver disease.

^j^History of intermittent claudication of presumed atherosclerotic origin.

^k^Classified according to the ACC/AHA/HRS guidelines.

^l^121/179 (67.6%) died during the hospital stay; 16/179 patients (8.9%) had severe in-hospital bleeding events, mainly ICH (*n* = 11/16), 31/179 (17.3%), had no identifiable clinical reason for withholding oral anticoagulation after the index stroke. Weighted SMDs > 0.10 are reported in bold.

Abbreviations: DOAC = direct oral anticoagulant; INR = international normalised ratio; SMD = standardised mean difference; VKA = vitamin K antagonist.

### Inverse probability weighting

After IPW, the weighted cohort (pseudopopulation) comprised 759 patients with cancer and 890 without. No missing data were present for propensity score variables. Balance diagnostics indicated adequate matching, with an SMD of 0.19 (<0.25) and a variance ratio of 1.01 (0.5–2.0).^14^ Visual inspection and covariate-balance plots confirmed the good overlap (Figures S1 and S2). Weighted baseline characteristics showed SMD < 0.10 across most variables (Table 1).

### Primary outcome

**Figure 2:**
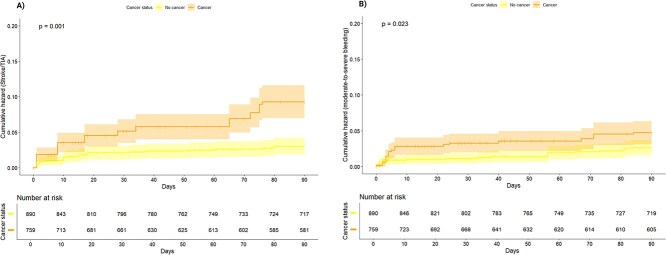
Kaplan–Meier cumulative estimates of (A) 90-day new ischaemic stroke or TIA and (B) 90-day moderate-to-severe bleeding by cancer status in the weighted cohort. *p*: log-rank test *P*-value. Dashed areas indicate 95% CIs.

In the unweighted cohort, 90-day ischaemic stroke or TIA occurred in 20 (8.1%) cancer and 37 (2.6%) non-cancer patients. After weighting and adjustment, the 90-day risk remained significantly higher in cancer (8.2% vs 2.8%; aHR 2.56; 95% CI, 1.59–4.13; *P* < .001) (Table 2; Figure 2A). In competing-risk analysis, the cumulative incidence differed by cancer status (Gray’s test χ^2^ = 23.6; *P* < .001), and Fine–Gray regression confirmed higher subdistribution hazard (SHR 2.97; 95% CI, 1.87–4.72; *P* < .001). Death as competing event was not different (*P* = .379) (Figure S3).

**Table 2:** Outcomes comparison.

	**Unweighted cohort**		**Weighted cohort**				
	**Cancer (*n* = 247)**	**No cancer (*n* = 1,402)**	**Cancer (*n* = 759)**	**No cancer (*n* = 890)**	**Statistical metric** ^**a**^	**Weighted difference [95% CI]**	***P* value**
***Primary outcome***							
**90-day new ischaemic stroke or TIA, *n* (%)**	20 (8.1)	37 (2.6)	62 (8.2)	25 (2.8)	Adjusted hazard ratio	2.56 [1.59–4.13]	**<.001**
***Secondary outcomes***							
**90-day mRS score distribution**					Adjusted odds ratio	1.29 [1.08–1.54]	**.005**
** No symptoms (score of 0), *n* (%)**	13 (5.3)	172 (12.3)	84 (11.1)	112 (12.6)			
** Symptoms without any disability (score of 1), *n* (%)**	33 (13.4)	251 (17.9)	85 (11.2)	163 (18.3)			
** Symptoms with mild disability (score of 2), *n* (%)**	36 (14.6)	205 (14.6)	128 (16.9)	150 (16.9)			
** Symptoms with mild-to-moderate disability (score of 3), *n* (%)**	48 (19.4)	221 (15.8)	145 (19.1)	140 (15.7)			
** Symptoms with moderate-to-severe disability (score of 4), *n* (%)**	42 (17.0)	178 (12.7)	121 (15.9)	110 (12.4)			
** Symptoms with severe disability (score of 5), *n* (%)**	19 (7.7)	97 (6.9)	60 (7.9)	57 (6.4)			
** Death (score of 6), *n* (%)**	56 (22.7)	278 (19.8)	136 (17.9)	158 (17.8)			
**90-day all-cause death, *n* (%)**	56 (22.7)	278 (19.8)	136 (17.9)	158 (17.8)	Adjusted hazard ratio	0.98 [0.78–1.24]	.883
**90-day vascular death, *n* (%)**	31 (12.6)	184 (13.1)	82 (10.8)	101 (11.3)	Adjusted hazard ratio	0.94 [0.70–1.26]	.677
***Safety outcomes***							
**90-day moderate-to-severe bleeding, *n* (%)**	16 (6.5)	39 (2.8)	33 (4.3)	21 (2.4)	Adjusted hazard ratio	1.82 [1.05–3.14]	**.033**
**90-day ICH, *n* (%)**	7 (2.8)	22 (1.6)	16 (2.1)	11 (1.2)	Adjusted hazard ratio	1.66 [0.77–3.58]	.194
**24-h haemorrhagic transformation, *n* (%)**	35 (14.2)	252 (18.0)	125 (16.5)	158 (17.8)	Adjusted risk difference (%)	0.2 [−3.8 to 4.2]	.931

Global Schoenfeld residual *P*-values: 90-day new ischaemic stroke or TIA = 0.422; 90-day vascular death = 0.550; 90-day all-cause death = 0.861; 90-day moderate-to-severe bleeding = 0.663; 90-day ICH = 0.224. Statistically significant *P*-values (<0.05) are reported in bold.

^a^Adjusted for baseline covariates that remained imbalanced (SMD > 0.10) after weighting (age, ethnicity, pre-stroke mRS, atrial fibrillation type, post-stroke anticoagulation strategy).

### Secondary outcomes

**Figure 3:**
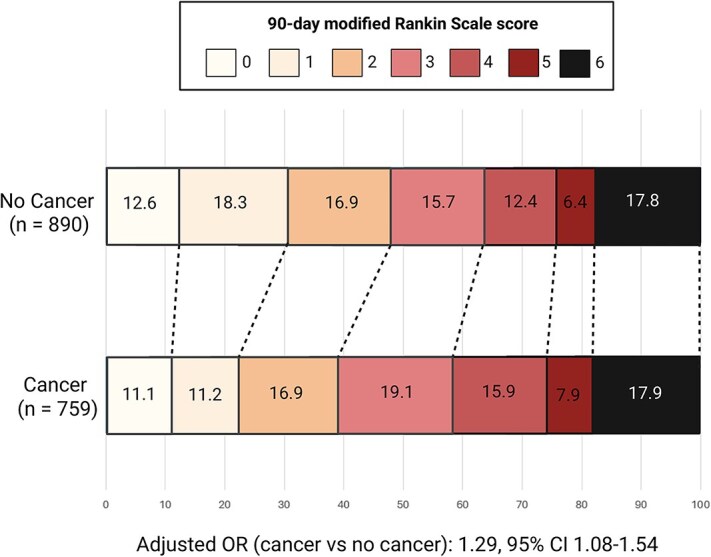
90-day mRS ordinal shift in the weighted cohort.

As shown in Table 2, patients with cancer had a significantly higher 90-day disability burden compared to those without cancer (adjusted odds ratio 1.29; 95% CI, 1.08–1.54; *P* = .005) (Figure 3), while 90-day all-cause and vascular mortality did not differ between groups.

### Safety outcomes

Moderate-to-severe bleeding occurred in 16 (6.5%) cancer and 39 (2.8%) non-cancer patients in the unweighted cohort. In the weighted analysis, cancer remained associated with higher 90-day bleeding risk (4.3% vs 2.4%; aHR 1.82; 95% CI, 1.05–3.14; *P* = .033) (Table 2; Figure 2B). Among cancer patients, 8/15 (53.3%) bleeding events were extracranial and 7/15 (46.7%) intracranial, with no differences vs non-cancer. The 24-h haemorrhagic transformation risk was similar (Table 2).

### Active cancer and cancer in remission versus no cancer

**Figure 4:**
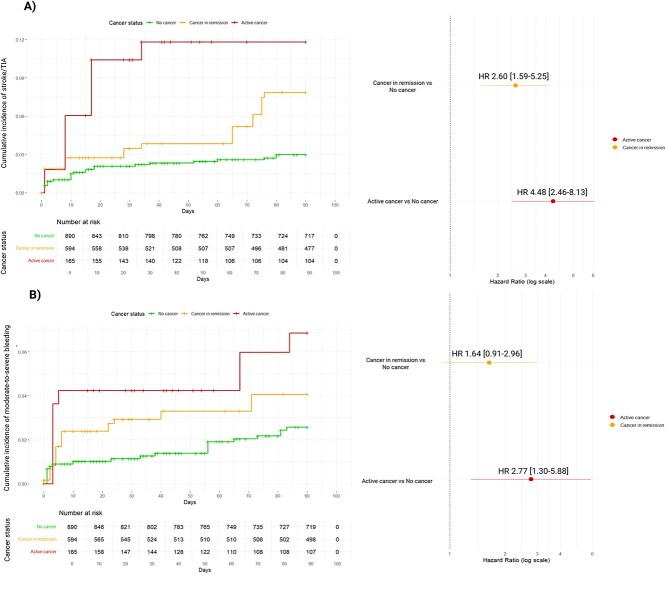
Kaplan–Meier cumulative estimates of (A) 90-day new ischaemic stroke or TIA and (B) 90-day moderate-to-severe bleeding in patients with active cancer, cancer in remission vs no cancer in the weighted cohort. Panels on the right indicate the HR (95% CIs) for 90-day new ischaemic stroke or TIA (A) and moderate-to-severe bleeding in respect to patients without cancer. Abbreviation: HR = hazard ratio.

Compared with non-cancer, patients with active cancer had higher 90-day risks of ischaemic stroke/TIA (HR 4.48; 95% CI, 2.46–8.13; *P* < .001) and bleeding (HR 2.77; 95% CI, 1.30–5.88; *P* = .008). Those with cancer in remission had elevated ischaemic risk (HR 2.60; 95% CI, 1.59–5.25; *P* = .001) but similar bleeding risk (HR 1.64; 95% CI, 0.91–2.96; *P* = .103) (Table S1; Figure 4).

### Haematological versus solid malignancies

In the weighted cohort, haematological cancers carried a higher 90-day risk of ischaemic recurrence (HR 3.06; 95% CI, 1.69–5.54; *P* = .001) and bleeding (HR 3.47; 95% CI, 1.57–7.70; *P* = .006) compared with solid tumours (Table S2; Figure S4).

## Discussion

In this multicentre cohort of patients with AF who experienced ischaemic stroke during continuous OAC, cancer was associated with a higher 90-day risk of recurrent ischaemic stroke or TIA, worse functional outcome and increased moderate-to-severe bleeding compared with patients without cancer. Patients with active cancer exhibited over a 4-fold higher risk of ischaemic recurrence and nearly 3-fold higher risk of moderate-to-severe bleeding, whereas those in remission showed an elevated risk of recurrent cerebral ischaemia only. Haematological malignancies were associated with markedly higher risks of both ischaemic and haemorrhagic events compared with solid tumours. Overall, these findings identify cancer as a key modifier of prognosis in AF patients with breakthrough stroke on OAC, underscoring the need for tailored secondary prevention strategies to balance competing ischaemic and bleeding risks.

The higher 90-day risk of recurrent ischaemic stroke or TIA among patients with cancer compared with those without suggests that malignancy amplifies thromboembolic burden despite OAC. This is biologically plausible, as cancer-related prothrombotic mechanisms, tumour-derived factors, anticancer therapies, platelet activation and coagulation cascade upregulation, promote thrombogenesis.^6–8^ Endothelial injury further enhances platelet adhesion via von Willebrand factor release, while many anticancer drugs alter CYP3A4 and P-glycoprotein activity, reducing DOAC exposure and efficacy.^9^ These mechanisms may attenuate OAC protection and explain recurrent ischaemic events.^8^ In our cohort, 89.1% resumed anticoagulation after stroke, without differences by cancer status. Although causality cannot be inferred, these findings suggest cancer may contribute both to index and recurrent strokes, reinforcing the need for integrated prevention strategies addressing cancer-related hypercoagulability.

Evidence on secondary prevention after breakthrough ischaemic stroke in cancer patients on OAC is scarce. Available data, mostly from embolic stroke of unknown source (ESUS) populations, indicate comparable recurrence rates between anticoagulants and antiplatelets. A retrospective study of 263 patients with active cancer showed no difference in thromboembolic recurrence between the 2 treatments.^15^ Similarly, the ARCADIA post hoc analysis (137 ESUS patients with prior cancer) and the NAVIGATE ESUS subanalysis reported similar efficacy between apixaban or rivaroxaban and aspirin.^16,17^ In our cohort, even patients with prior (remitted) cancer had a higher recurrence risk than non-cancer patients, though lower than those with active malignancy. This gradient suggests that the prothrombotic state may persist beyond active disease, warranting long-term vigilance and optimisation of preventive therapy.

Regarding safety outcomes, active cancer patients had an approximately 3-fold higher 90-day risk of moderate-to-severe bleeding, mostly extracranial, mainly driven by patients with haematological malignancies, who showed the highest risk for both moderate-to-severe bleeding and ischaemic recurrence. This pattern aligns with previous evidence^18^ and likely reflects the profound haemostatic dysregulation characteristic of haematological cancers, including coagulopathy, thrombocytopenia and, in severe cases, evolution towards disseminated intravascular coagulation.^19–21^ Furthermore, haematological cancer therapies may amplify both venous and arterial thromboembolic risk or accelerate atherosclerotic processes,^19–21^ creating a particularly unfavourable balance between ischaemic and haemorrhagic complications in anticoagulated patients.^22,23^ In contrast, solid tumours may contribute to bleeding risk through both local and systemic mechanisms: tumour invasion and neovascularisation generate fragile vasculature, while systemic coagulopathy, treatment-related thrombocytopenia and endothelial dysfunction impair haemostasis.^24^ In anticoagulated patients, these alterations, together with drug–drug interactions and fluctuating renal or hepatic function, can modify anticoagulant metabolism, narrowing the therapeutic window and further predisposing to bleeding.^22,23^

The main strength of this study lies in the rigorous data verification and systematic quality checks of the ASPERA-R database, its large sample size and the use of advanced statistical methods providing robust outcome estimates. However, several limitations must be acknowledged. First, despite IPW adjustment, residual confounding cannot be excluded given the observational design. Second, the retrospective nature may have introduced selection or measurement bias and precludes causal inference; some cases managed outside the stroke unit or with alternative discharge diagnoses may have been missed. Third, DOAC plasma levels were available for only ~ 20% of patients, limiting assessment of treatment exposure; estimates based on clinical data may have led to misclassification, and time on therapeutic INR was unavailable for VKA users. Fourth, resumption of OAC after the index stroke was incomplete: among those who did not restart anticoagulation (10.9%), most died during the index hospitalisation or had severe in-hospital bleeding, and in a minority no clear clinical reason was identifiable. This may have influenced both thromboembolic and bleeding outcomes and represents an additional potential source of residual confounding. Fifth, 6.9% of patients with incomplete data were excluded, although attrition appeared nondifferential, selection bias remains possible. Sixth, although receipt of cancer-directed therapy and the presence of recurrence or metastasis were collected in binary form to classify active cancer, detailed information on cancer stage, specific therapeutic regimens and extent of metastatic disease was not available, limiting assessment of how these features may influence OAC exposure and outcomes. This is relevant because bleeding risk (elevated in cancers such as melanoma or renal cell carcinoma) and thromboembolic risk (notably increased in advanced lung or pancreatic cancer) vary substantially by tumour type.^8,24^ Furthermore, assessing the effect of specific cancer subtypes on ischaemic recurrence is inherently challenging, as this would require very large sample sizes to support adequately powered subgroup analyses. Lastly, advanced disease, chemotherapy-related cytopenias, frailty and cachexia, key modifiers of thrombotic and haemorrhagic risk, were not systematically captured and may have attenuated, though not reversed, the observed associations. In addition, as this study is a predefined secondary analysis of the ASPERA-R registry, which was designed to capture major cardiovascular effectiveness outcomes and bleeding, systematic data on venous thromboembolism were not collected, thereby preventing a reliable risk assessment despite it may influence antithrombotic management in patients with cancer. Moreover, undiagnosed cancers in the no-cancer group cannot be ruled out, as systematic screening was not performed. Similarly, limited data on metastatic disease precluded reliable assessment of its impact. Finally, since ~ 60% of patients were enrolled in Italy and most were non-Hispanic White, generalisability to other populations may be limited.

In conclusion, cancer significantly alters the prognosis of patients experiencing ischaemic stroke despite OAC, increasing short-term risks of both recurrent ischaemia and major bleeding, particularly in those with active or haematological malignancies. These results emphasise the delicate interplay between hypercoagulability, treatment-related haemostatic changes and anticoagulant safety, underscoring the need for refined risk assessment and individualised management. Future prospective studies should clarify the mechanisms underlying anticoagulation failure in cancer and define evidence-based strategies for secondary prevention to support precision antithrombotic therapy and improve outcomes in this high-risk population.

## Supplementary Material

aakag015_SUPPLEMENTAL_ASPERA_CANCER_final

## Data Availability

The dataset supporting this study will be made available upon reasonable request to the corresponding author.
