# Supplementary material for: Impact of cancer on outcomes following breakthrough ischaemic stroke on oral anticoagulants for atrial fibrillation: insights from the ASPERA-R study
Source: Eur Stroke J. 2026 Feb 27;11(2):aakag015. doi: 10.1093/esj/aakag015 (PMC12947708; doi:10.1093/esj/aakag015)
Supplement: aakag015_SUPPLEMENTAL_ASPERA_CANCER_final [file aakag015_supplemental_aspera_cancer_final.docx]

**Supplemental material**

**Figure S1. Propensity score distribution in the unweighted and weighted cohort**

**
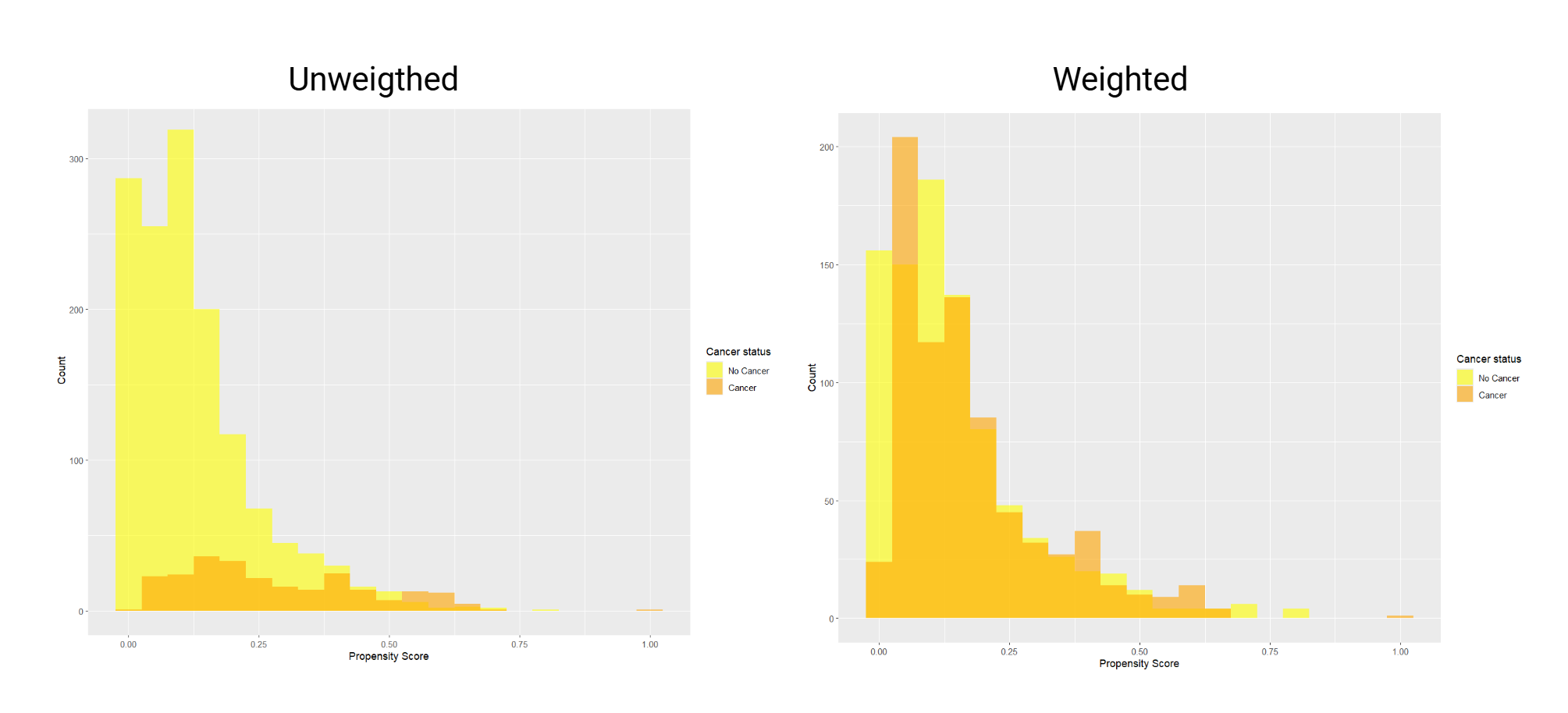
**

**Figure S2. Love plot of covariate balance in the unweighted and weighted cohort**

**
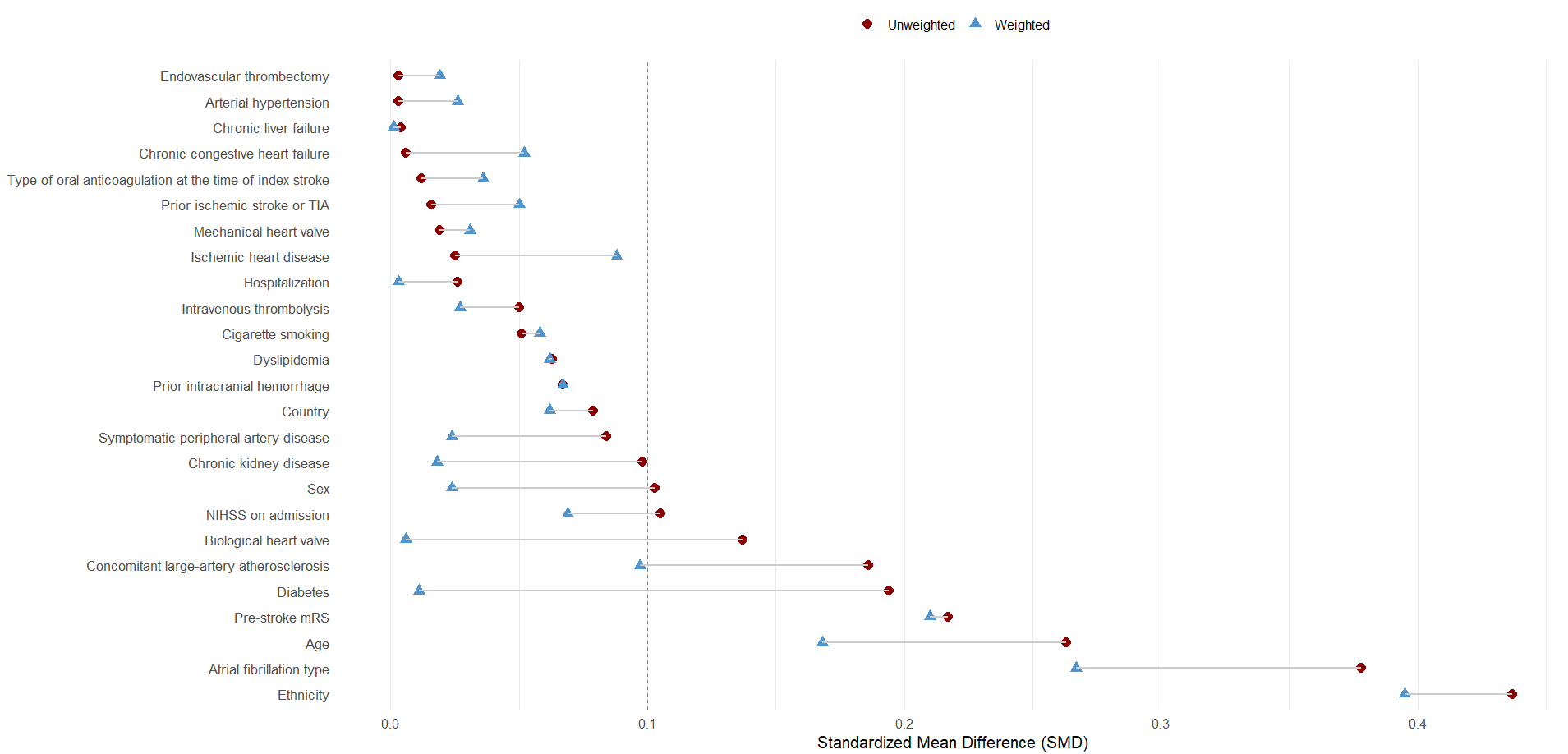
**

**Figure S3.** **Competing risk analysis for 90-day new ischemic stroke or TIA and 90-day all-cause death (Fine-Gray model)**


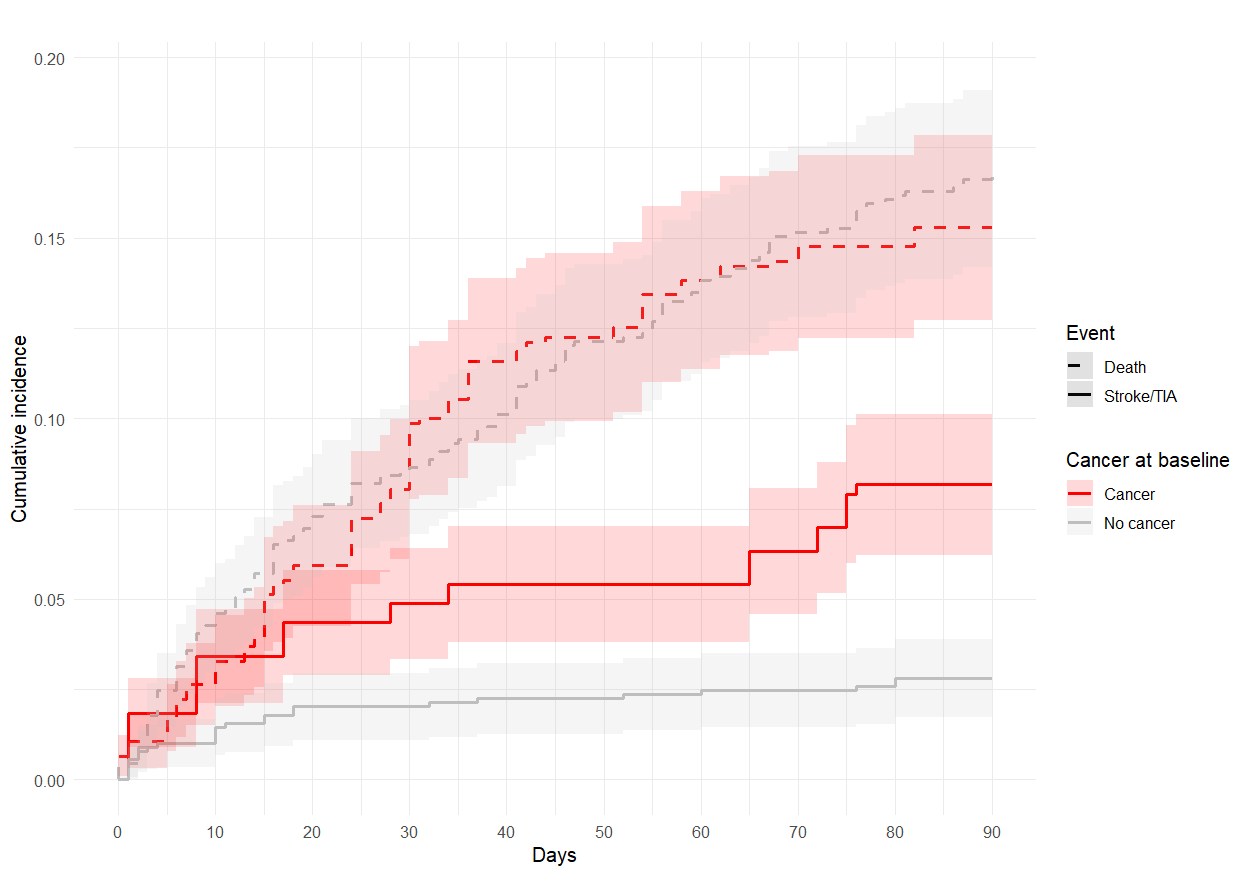


*Shaded areas indicate 95% confidence intervals.*

**Figure S4. Kaplan-Meier cumulative estimates of A) 90-day new ischemic stroke or TIA and B) 90-day moderate-to-severe bleeding in patients with hematological versus solid malignancies in the weighted cohort**

**
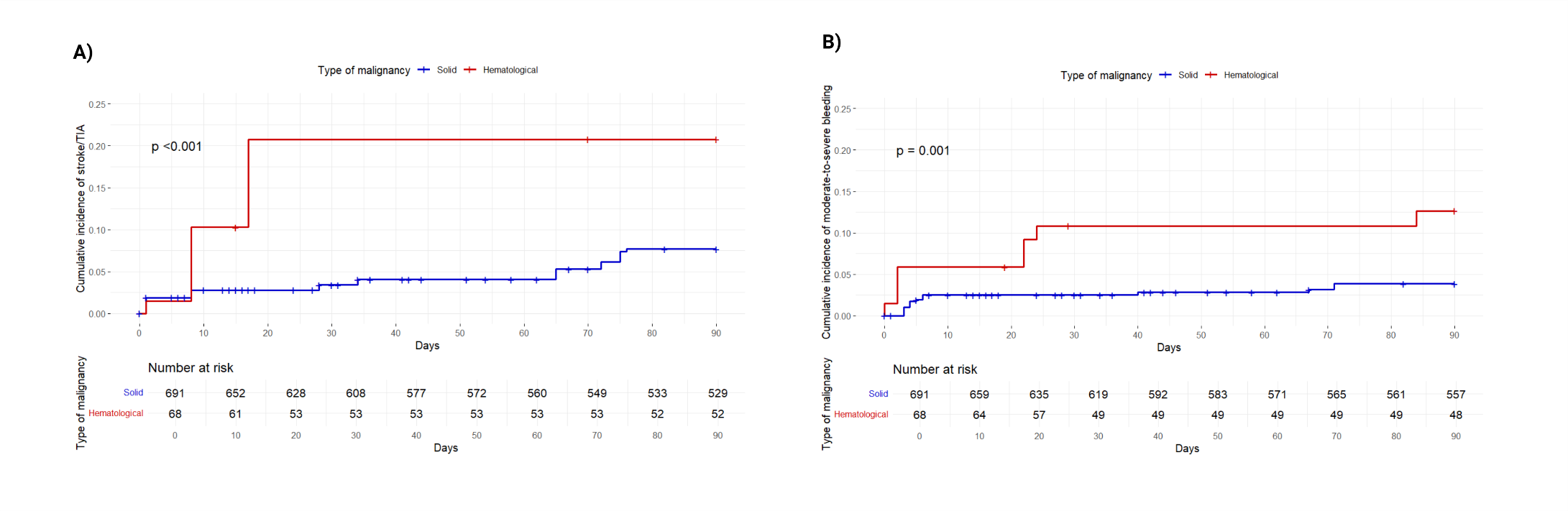
**

*p: log-rank test p-value.*

**Table S1. Comparison of 90-day new ischemic stroke or TIA and 90-day moderate-to-severe bleeding between patients with active cancer, cancer in remission and no cancer in the weighted cohort**

|  | | **N/total (%)** | **HR (vs no cancer)** | **95% CI** | **P value** |
| --- | --- | --- | --- | --- | --- |
| 90-day new ischemic stroke and other vascular events | No cancer | 25/890 (2.8) | 1 | Ref. | - |
|  | Cancer in remission | 43/594 (7.2) | 2.60 | 1.59-5.25 | **0.001** |
|  | Active cancer | 19/165 (11.5) | 4.48 | 2.46-8.13 | **<0.001** |
| 90-day moderate-to-severe bleeding | No cancer | 21/890 (2.4) | 1 | Ref. | - |
|  | Cancer in remission | 23/594 (3.9) | 1.64 | 0.91-2.96 | 0.103 |
|  | Active cancer | 10/165 (6.1) | 2.77 | 1.30-5.88 | **0.008** |

*Abbreviations. CI: confidence intervals; HR: hazard ratio. Statistically significant (<0.05) p-values are reported in bold.*

**Table S2. Comparison of 90-day new ischemic stroke or TIA and 90-day moderate-to-severe bleeding between patients with hematologic versus solid malignancies in the weighted cohort**

|  | | **N/total (%)** | **HR (vs solid malignancies)** | **95% CI** | **P value** |
| --- | --- | --- | --- | --- | --- |
| 90-day new ischemic stroke or TIA | Solid | 48/691 (6.9) | 1 | Ref. | - |
|  | Hematological | 14/68 (20.6) | 3.06 | 1.69-5.54 | **0.001** |
| 90-day moderate-to-severe bleeding | Solid | 25/666 (3.6) | 1 | Ref. | - |
|  | Hematological | 8/60 (11.8) | 3.47 | 1.57-7.70 | **0.006** |

*Abbreviations. CI: confidence intervals; HR: hazard ratio. Statistically significant (<0.05) p-values are reported in bold.*

**Appendix S1. Distribution of patients by country in the ASPERA-R study**

| **Country** | **Enrolling center(s)** | **No. of patients/total no. included in the ASPERA-R(%)** | **No. of patients/total no. included in the study analysis (%)** |
| --- | --- | --- | --- |
| Italy | - Stroke Unit and Neurology Unit, S.S. Filippo e Nicola Hospital, Avezzano. - Stroke Unit, Maggiore Hospital, Bologna. - Neurology Unit, IRCCS Policlinico S.Orsola-Malpighi, Bologna. - Stroke Unit, IRCCS Policlinico Universitario Agostino Gemelli, Rome. - Stroke Unit – Neurology Unit, S.Maria delle Croci Hospital, Ravenna. - Stroke Unit, ASST Grande Ospedale Metropolitano Niguarda, Milano. - Stroke Unit, Luigi Sacco Hospital, Milano. - Stroke Unit, Policlinico San Matteo, Pavia. - Stroke Unit – Neurology Unit, Vito Fazzi Hospital, Lecce. - Stroke Unit, Fabrizio Spaziani Hospital, Frosinone. - Stroke Unit, S.Maria della Misericordia Hospital, Perugia. - Stroke Unit, Azienda Ospedaliera Ospedali Riuniti Villa Sofia – Cervello, Palermo. - Stroke Unit, Arcispedale Santa Maria Nuova, Reggio Emilia. - Stroke Unit, AORN Cardarelli Hospital, Napoli. - Stroke Unit, Azienda Ospedaliero Universitaria Careggi, Firenze. - Stroke Unit, S.Maria della Misericordia Hospital, Udine. - Stroke Unit, Ospedale Riuniti di Ancona, Ancona. - Stroke Unit, Maggiore Hospital, Crema. - Stroke Unit – Neurology Unit, “Di Venere” Hospital, Bari. | 1010/1772 (57.0) | 989/1649 (60.0) |
| France | - Stroke Unit, Université Côte d’Azur, Nice. | 79/1772 (4.5) | 78/1649 (4.7) |
| Germany | - Stroke Unit – Neurology Unit, Martin Luther University, Halle-Wittenberg. - Stroke Unit, Saarland University Hospital, Homburg. | 67/1772 (3.8) | 43/1649 (2.6) |
| Spain | - Stroke Unit, Hospital Universitario La Paz, Madrid | 81/1772 (4.6) | 80/1649 (4.9) |
| Portugal | - Stroke Unit, São José Hospital, Lisbon. - Stroke Unit, Hospital de Santa Maria, Lisbon. | 124/1772 (7.0) | 101/1649 (6.1) |
| United Kingdom | - Stroke Unit, Charing Cross Hospital, London. - Stroke Unit, St George University Hospital, London. - Stroke Unit, North Bristol NHS Trust, Bristol. | 180/1772 (10.2) | 178/1649 (10.8) |
| Croatia | - Stroke Unit, Sveti Duh University Hospital, Zagreb. | 35/1772 (19.7) | 32/1649 (19.4) |
| Egypt and Saudi Arabia | - Stroke Unit – Neurology Unit, Aim Shams University Hospital, Cairo. - Stroke Unit, Armed Forces Medical Complex Kobry El Kobba, Cairo. - Stroke Unit, Cairo University Hospital, Cairo. - Stroke Unit, Assiut University Hospital, Asyut. - Stroke Unit – Neurology Unit, King Fahd Hospital, Riyahd. | 196/1772 (11.1) | 148/1949 (9.0) |

**Appendix S2. List of baseline variables collected in the ASPERA-R study**

| **Variable** | **Mandatory** | **Notes** |
| --- | --- | --- |
| **Demographics** | | |
| Date of index ischemic stroke on oral anticoagulation | Yes | - |
| Hospitalization (Yes/No) | Yes | - |
| Hospitalization setting   - Stroke unit - Intensive care unit - Other hospital unit - Emergency department only | Yes | - |
| Date of admission | Yes | - |
| Sex   - Male - Female - Other | Yes | - |
| Ethnicity   - Non-Hispanic white - Hispanic white - Black - Asian - Other | Yes | - |
| Date of birth | Yes | - |
| Weight – kg | No | - |
| Height – cm | No | - |
| **Risk factors** | | |
| Current cigarette smoking (Yes/No) | Yes | Consumption of ≥1 cigarette per day over the last year |
| Arterial hypertension (Yes/No) | Yes | Blood pressure of ≥140/90 mmHg at least twice before stroke or already under treatment with antihypertensive drugs |
| Dyslipidemia (Yes/No) | Yes | History of total blood cholesterol levels >220 mg/dL and/or total triglycerides levels >130 mg/dL and/or current used lipid-lowering drugs |
| Diabetes (Yes/No) | Yes | History of fasting glucose >126 mg/dL or the current use of hypoglycemic medications |
| Ischemic heart disease (Yes/No) | Yes | History of myocardial infarction, angina or prior evidence of coronary disease on coronary angiography |
| Chronic congestive heart failure (Yes/No) | Yes | History of stage C (structural heart disease and current or past history of heart-failure symptoms) or stage D (refractory symptoms that interfere with daily life or recurrent hospitalization despite targeted guideline-directed medical therapy) chronic heart failure |
| Chronic kidney disease (Yes/No) | Yes | History of estimated creatinine clearance of less than 60 for 3 months or more (including dialysis) |
| Chronic liver failure (Yes/No) | Yes | History of cirrhosis or end-stage liver disease |
| Symptomatic peripheral artery disease (Yes/No) | Yes | History of intermittent claudication of presumed atherosclerotic origin |
| Prior ischemic stroke or TIA (Yes/No) | Yes | - |
| Prior intracranial hemorrhage (Yes/No) | Yes | - |
| History of malignancy (Yes/No) | Yes | - |
| Type of malignancy   - Active - In remission | Yes | We defined active malignancy as (1) a diagnosis of cancer that occurred within 6 months of the index event or during hospitalization, (2) cancer treatment with radiotherapy, chemotherapy or surgery or a combination of them within 6 months of the index event, (3) a previous history of malignancy and a diagnosis of recurrence or metastasis within 6 months of the index event. In remission was defined as a previous history of malignancy in the absence of active cancer criteria. |
| Site of malignancy   - Gastrointestinal - Lung - Genitourinary - Breast - Hematological - Skin - Other | Yes | - |
| Metastatic malignancy? (Yes/No) | Yes | - |
| Metastasis location   - Lymph nodes - Liver - Lung - Bones - Brain/spine/meningeal - Other | Yes | - |
| Atrial fibrillation type   - Paroxysmal - Persistent - Long-standing persistent - Permanent | Yes | According to the ACC/AHA/HRS guidelines classification |
| History of valvular heart disease (multiple choice)   - Sever mitral stenosis - Sever aortic stenosis - Severe mitral insufficiency - Severe aortic insufficiency - Mechanical heart valve - Biological heart valve | Yes | - |
| Pacemaker (Yes/No) | Yes | - |
| Left atrial volume index at transthoracic echocardiography – mL/m^2^ | No | - |
| Left ventricle end-diastolic volume at transthoracic echocardiography – mL | No | - |
| Left ventricle end-systolic volume at transthoracic echocardiography – mL | No | - |
| Left ventricle ejection fraction at transthoracic echocardiography – mL | No | - |
| **Drugs history** | | |
| Type of oral anticoagulant ongoing at the time of the index ischemic stroke   - Vitamin K antagonist - Direct oral anticoagulant | Yes | - |
| Type of direct oral anticoagulant   - Apixaban - Rivaroxaban - Edoxaban - Dabigatran | Yes | - |
| Type of vitamin K antagonist   - Warfarin - Acenocoumarol - Other | Yes | - |
| Time from last direct oral anticoagulant intake to admission   - <12 hours - 12-24 hours - 24-48 hours | Yes | - |
| Direct oral anticoagulant plasma levels or anti-factor Xa activity available on admission | Yes | - |
| Direct oral anticoagulant plasma levels (ng/mL) or anti-factor Xa activity on admission | Yes | - |
| Direct oral anticoagulant plasma levels or anti-factor Xa activity on admission   - Below range - Within range - Above range | Yes | In respect to the range locally determined for therapeutic anticoagulation |
| Direct oral anticoagulant at reduced dose at the time of the index ischemic stroke (Yes/No) | Yes | Apixaban 2.5mg BID, dabigatran 75mg BID, edoxaban 30mg daily, rivaroxaban 15mg daily |
| Direct oral anticoagulant reduced dose appropriate (Yes/No) | Yes | Reduced dose of apixaban was considered appropriate if 2 of 3 factors were present: 1) Age ≥80 years; 2) serum creatinine ≥1.5 mg/dL; 3) Weight ≤60 kg OR if apixaban is co-administered with combined P-gp and strong CYP3A4 inhibitors (e.g., ketoconazole, itraconazole, ritonavir). Reduced dose of dabigatran was considered appropriate if creatinine clearence 15-30 mL/min OR, creatinine clearence 30-50 mL/min with concomitant dronedarone or ketoconazole. Reduced dose of edoxaban was considered appropriate if creatinine clearence 15-50 mL/min. Reduced dose of rivaroxaban was considered appropriate if creatinine clearence ≤50 mL/min |
| Antihypertensive drugs on admission (Yes/No) | Yes | - |
| Lipid-lowering drugs on admission (Yes/No) | Yes | - |
| Antidiabetic drugs on admission (Yes/No) | Yes | - |
| Rhythm control drugs on admission (Yes/No) | Yes | - |
| Type of rhythm control drugs on admission   - Class I – Sodium channel blockers - Class II – Beta-blockers - Class III – Potassium channel blockers - Class IV – Calcium channel blockers - Class V – Miscellaneous agents (i.e., digoxin, ivabradine, adenosine) | Yes | According to the Vaughan Williams classification of antiarrhythmic drugs |
| Drugs potentially interfering with oral anticoagulation at the time of the index stroke (multiple choice)   - Itraconazole - Ketoconazole - Clarithromycin - Lopinavir - Indinavir - Ritonavir - Telaprevir - Voriconazole - Any H2 inhibitor (i.e., cimetidine) - Any proton pump inhibitor (i.e. omeprazole, pantoprazole) - Doxorubicin - Vinblastine - Carbamazepine/ Oxcarbazepine - Phenytoin - Phenobarbital - Rifampin - Levetiracetam - Valproic acid - Dexamethasone - Tocilizumab |  | - |
| Antiplatelet therapy on admission (Yes/No) | Yes | - |
| Type of antiplatelet therapy on admission (multiple choice)   - Aspirin - Clopidogrel - Ticagrelor - Ticlopidine - Other | Yes | - |
| Antihypertensive drugs on admission (Yes/No) | Yes | - |
| **Clinical characteristics** | | |
| Modified Rankin Scale (mRS) score before the index ischemic stroke | Yes | - |
| Type of ischemic stroke onset   - Known onset - Wake-up stroke - Unwitnessed stroke | Yes |  |
| National Institute of Health Stroke Scale (NIHSS) score on admission (prior to any acute reperfusion therapy) | Yes | - |
| Acute ischemic stroke symptoms (multiple choice)   - Motor weakness - Aphasia - Dysarthria - Sensory defect - Visual field defect - Diplopia - Vertigo - Loss of balance - Hemineglect - Other | No | - |
| Clinical classification of the acute ischemic stroke   - Total anterior circulation stroke (TACS) - Partial anterior circulation stroke (PACS) - Posterior circulation syndrome (PoCS) - Lacunar stroke (LACS) | Yes | According to the Oxfordshire Stroke Project Classification |
| Admission systolic blood pressure - mmHg | No | - |
| Admission diastolic blood pressure – mmHg | No | - |
| Admission Glasgow Coma Scale (GCS) score | No | - |
| Presence of competing stroke etiology other than cardioembolism (Yes/No) | Yes | - |
| Type of competing stroke etiology other than cardioembolism   - Lacunar - Large artery atherosclerosis - Other determined etiology | Yes | According to the Trial of Org 10172 in the Acute Stroke Treatment (TOAST) classification system |
| Type of other determined etiology (specify) | Yes | - |
| Competing cardioembolic mechanisms other than atrial fibrillation (Yes/No) | Yes | - |
| Type of competing cardioembolic mechanisms other than atrial fibrillation (multiple choice)   - Reduced left ventricle ejection fraction (30-40%) - Severely reduced left ventricle ejection fraction (< 30%) - Left ventricle thrombus - Endocarditis - Mechanical heart valve - Biological heart valve - Atrial myxoma - Fibroelastoma - Other cardiac tumors - Patent foramen ovale (PFO) - Interventricular defect (IVD) - Left ventricular aneurysm - Left atrial aneurysm - Other cardiac congenital alterations | Yes | - |
| Intravenous thrombolysis (Yes/No) | Yes | - |
| Endovascular thrombectomy (Yes/No) | Yes | - |
| Onset-to-needle time – minutes | Yes | - |
| Onset-to-groin time – minutes | Yes | - |
| Hemorrhagic infarction   - Yes - asymptomatic - Yes – symptomatic - No | Yes | Symptomatic hemorrhagic infarction was defined according to SITS-MOST as a neurologic deterioration of ≥4 NIHSS points or leading to death within 24 hours |
| Hemorrhagic infarction type (multiple choice)   - HI1: Scattered small petechiae, no mass effect - HI2: Confluent petechiae, no mass effect - PH1: Hematoma within infarcted tissue, occupying < 30%, no substantive mass effect - PH2: Hematoma occupying 30% or more of the infarcted tissue, with obvious mass effect PH remote from infarcted brain tissue - rPH: Remote parenchymal hematoma - Intraventricular hemorrhage - Subarachnoid hemorrhage - Subdural hemorrhage | Yes | According to the Heidelberg classification system |
| National Institute of Health Stroke Scale (NIHSS) score at 24 hours | No | - |
| **Neuroimaging information** | | |
| Type of brain neuroimaging performed on admission   - Non-contrast computed tomography - Magnetic resonance imaging - Both | Yes | - |
| Type of brain neuroimaging performed at 24 hours follow-up   - Non-contrast computed tomography - Magnetic resonance imaging - Both - None | Yes | - |
| Type of brain vessel imaging performed (multiple choice)   - Computed tomography angiography - extracranial vessels - Computed tomography angiography - intracranial vessels - Magnetic resonance angiography - extracranial vessels - Magnetic resonance angiography - intracranial vessels - Color Doppler ultrasonography - extracranial vessels - Color Doppler ultrasonography - intracranial vessels - X-ray angiography - None | Yes | - |
| Large vessel occlusion (Yes/No/Unknown) | Yes | - |
| Large vessel occlusion site (multiple choice)   - Middle cerebral artery - M1 - Middle cerebral artery - M2 - Middle cerebral artery - More distal than M2 - Tandem occlusion - Anterior cerebral artery - Posterior cerebral artery - Basilar artery - Internal carotid artery - Vertebral artery | Yes | - |
| Baseline modified Thrombolysis in Cerebral Infarction score   - Grade 0: no perfusion - Grade 1: antegrade reperfusion past the initial occlusion, but limited distal branch filling with little or slow distal reperfusion - Grade 2a: antegrade reperfusion of less than half of the occluded target artery previously ischemic territory (i.e., in one major division of the middle cerebral artery and its territory) - Grade 2b: antegrade reperfusion of more than half of the previously occluded target artery ischemic territory (i.e., in two major divisions of the MCA and their territories) - Grade 2c: near complete perfusion except for slow flow or distal emboli in a few distal cortical vessels - Grade 3: complete antegrade reperfusion of the previously occluded target artery ischemic territory, with absence of visualized occlusion in all distal branches | Yes | - |
| Post-endovascular thrombectomy modified Thrombolysis in Cerebral Infarction score   - Grade 0: no perfusion - Grade 1: antegrade reperfusion past the initial occlusion, but limited distal branch filling with little or slow distal reperfusion - Grade 2a: antegrade reperfusion of less than half of the occluded target artery previously ischemic territory (i.e., in one major division of the middle cerebral artery and its territory) - Grade 2b: antegrade reperfusion of more than half of the previously occluded target artery ischemic territory (i.e., in two major divisions of the MCA and their territories) - Grade 2c: near complete perfusion except for slow flow or distal emboli in a few distal cortical vessels - Grade 3: complete antegrade reperfusion of the previously occluded target artery ischemic territory, with absence of visualized occlusion in all distal branches | Yes | - |
| Presence of extracranial internal artery stenosis ≥50% ipsilateral to the acute ischemic stroke lesion (Yes/No) | Yes | - |
| Degree of extracranial internal artery stenosis ≥50% ipsilateral to the acute ischemic stroke lesion   - 50-69% - 70-79% - 80-99% - Occlusion (100%) | Yes | - |
| Presence of extracranial vertebral artery stenosis ≥50% ipsilateral to the acute ischemic stroke lesion (Yes/No) | Yes | - |
| Degree of extracranial vertebral artery stenosis ≥50% ipsilateral to the acute ischemic stroke lesion   - 50-69% - 70-79% - 80-99% - Occlusion (100%) | Yes | - |
| Presence of intracranial artery stenosis (Yes/No) | Yes | - |
| Site of intracranial artery stenosis (multiple choice)   - Middle cerebral artery - Anterior cerebral artery - Posterior cerebral artery - Basilar artery - Internal carotid artery - Vertebral artery | Yes | - |
| Presence of symptomatic intracranial artery stenosis (Yes/No) | Yes | - |
| **Laboratory tests** | | |
| International Normalized Ratio (INR) | Yes | - |
| aPTT – seconds | No | - |
| PT – seconds | No | - |
| Red blood cells – millions/mm^3^ | No | - |
| White blood cells – millions/mm^3^ | No | - |
| Lymphocytes – millions/mm^3^ | No | - |
| Neutrophiles – millions/mm^3^ | No | - |
| Platelets – thousands/mm^3^ | No | - |
| Hemoglobin – mg/dL | No | - |
| Admission blood glucose levels – mg/dL | No | - |
| Fasting blood glucose levels – mg/dL | No | - |
| C reactive protein – mg/dL | No | - |
| Creatinine – mg/dL | No | - |
| Creatinine clearance – mL/min | No | - |
| Glycate hemoglobin – mmol/L | No | - |
| Total cholesterol – mg/dL | No | - |
| High density lipoprotein (HDL) – mg/dL | No | - |
| Low density lipoprotein (LDL) – mg/dL | No | - |
| Triglycerides – mg/dL | No | - |
| ALT/GPT – U/L | No | - |
| AST/GOT – U/L | No | - |
| Gamma-GT – U/L | No | - |
| Total bilirubin – mg/dL | No | - |
